# Targeting breast cancer stem cells directly to treat refractory breast cancer

**DOI:** 10.3389/fonc.2023.981247

**Published:** 2023-05-12

**Authors:** Liping Pan, Juan Han, Ming Lin

**Affiliations:** ^1^ Wuhan Center for Clinical Laboratory, Wuhan, China; ^2^ Department of Pediatrics, Union Hospital, Tongji Medical College, Huazhong University of Science and Technology, Wuhan, China

**Keywords:** breast cancer, immunotherapy, cancer stem cells, immunoresistance, oncology

## Abstract

For patients with refractory breast cancer (BC), integrative immunotherapies are emerging as a critical component of treatment. However, many patients remain unresponsive to treatment or relapse after a period. Different cells and mediators in the tumor microenvironment (TME) play important roles in the progression of BC, and cancer stem cells (CSCs) are deemed the main cause of relapse. Their characteristics depend on their interactions with their microenvironment as well as on the inducing factors and elements in this environment. Strategies to modulate the immune system in the TME of BC that are aimed at reversing the suppressive networks within it and eradicating residual CSCs are, thus, essential for improving the current therapeutic efficacy of BC. This review focuses on the development of immunoresistance in BCs and discusses the strategies that can modulate the immune system and target breast CSCs directly to treat BC including immunotherapy with immune checkpoint blockades.

## Introduction

Breast cancer (BC) is one of the most commonly diagnosed cancer types among women globally, with 2.26 million new cases diagnosed in 2020, according to the World Cancer Research Fund. It is the second leading cause of cancer death in women living in developed countries. However, with advances in detection and treatment, death rates from BC have been declining, and more recent advancements in BC immunotherapy have opened new avenues for reducing the death rate further. BC could be classified into five distinct subtypes: luminal A, luminal B, basal-like, normal breast-like, and HER-2 enriched (1) and traditionally, mammography has been used as a gold standard in the screening of BC (2). Most women with breast cancer in stages I, II, or III are treated with surgery, often followed by radiation therapy, while for women with stage IV breast cancer, systemic drug therapies are the main treatments (3). Although treatment with trastuzumab and other human epidermal growth factor receptor 2 (HER2)-directed therapies are associated with significant efficacy, only patients with the highest levels of HER2 expression, representing approximately 20% of patients with BC, have the potential to respond. Moreover, many patients expressing high levels of HER2 progress or relapse despite receiving the best HER2-directed treatments, and thus require novel treatment approaches. Additionally, for patients with estrogen receptor-positive (ER+) or progesterone receptor-positive (PR+) BC who are refractory to endocrine therapy, or patients who have triple negative BC, targeted therapeutic options remain quite limited. Consequently, new therapeutic strategies for BC are needed to improve clinical outcomes for patients with BC, particularly those with advanced disease. Other immunotherapies are currently being tested in BC clinical trials and several have already shown impressive results.

## Interactions between breast cancer stem cells and the immune system

**Figure 1:**
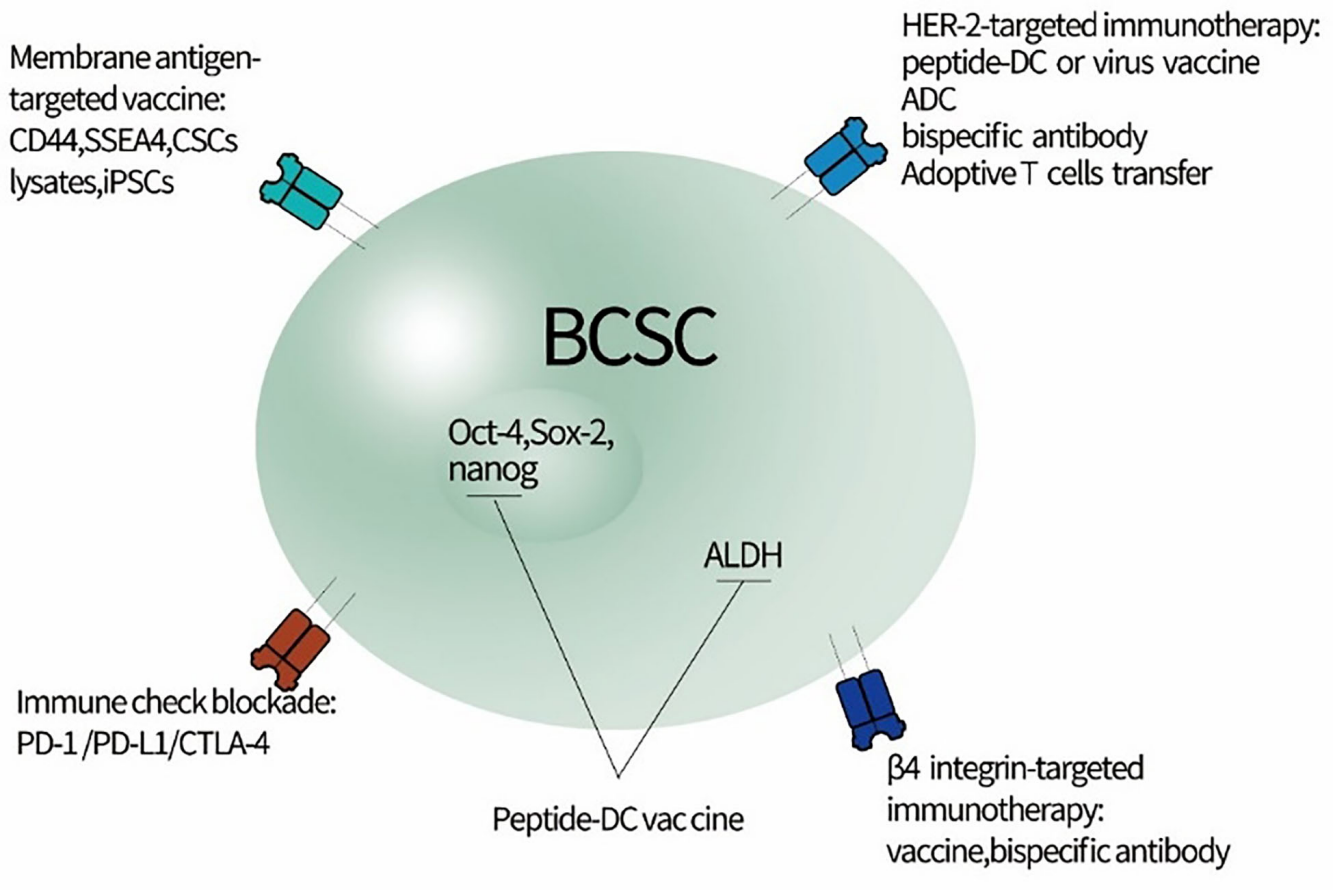
Potential strategy for directly targeting BCSCs to relieve immunoresistance. BCSC, breast cancer stem cells; iPSCs, induced pluripotent stem cells; CSCs, cancer stem cells; ADC, antibody-drug conjugate; SSEA4, stage-specific embryonic antigen 4.

The mammary gland stroma and, in particular, immune cells play a critical role during the organogenesis of the gland (4). Innate immune cells are important positive regulators of the mammary gland terminal end bud (TEB) elongation and branching (5). Macrophages and eosinophils drive TEB invasion within the mammary fat pad environment, and mast cells help the branching process by releasing serine-proteases (6). Csf1op/op mice, with a homozygous Csf1 mutation, have a severe reduction of macrophages. Mammary stem cell (MaSC) transplantation assays into Csf1op/op macrophage-depleted mammary fat pads showed a compromised epithelial regeneration ability, demonstrating the macrophage supportive function of MaSCs. Additionally, macrophage infiltration during mammary gland involution is critical for an adequate clearing of dead epithelial cells in the involuting gland and should not affect the stem cell pool for future pregnancy cycles. Macrophages fluctuate during mammary gland development, reaching higher levels during lactation-involution and tumorigenesis (7). These mammary gland macrophages secrete natural interferon alpha (IFN-α) and mediate a differential effect on luminal progenitor/mature cells compared to the MaSCs. While MaSCs are protected from the suppressive intrinsic effects of IFN-α (cell cycle arrest, apoptosis, and differentiation), the luminal cells are highly sensitive to terminal differentiation upon IFN-α cellular response (7) (**Figure 1**). Therefore, the interplay of immune cells diverges between stem cells and differentiated cells, and this is critical for mammary gland repopulation.

The adaptive immune system can also regulate luminal differentiation in the mammary gland (**Figure 1**).

While multiple studies have revealed the important roles played by immune cells and other stromal components in the mammary gland, the MaSC niche remained elusive until very recently. Gli2+ stromal cells have been shown to form a supportive niche for MaSCs, supplying them with insulin-like growth factor 1 and Wnt2B in response to the secretion of estrogens and growth hormones (8). Importantly, another very recent study has identified a macrophageal niche for MaSCs, wherein DLL1+ MaSCs directly interact with Notch in macrophages (9). This direct interaction triggers the expression of Wnt factors in macrophages, which are secreted and feed back to MaSCs maintaining their stemness (**Figure 1**). This further demonstrates the critical interplay between immune cells and stem cells during mammary gland development.

## Targeting the tumor microenvironment

BC comprises a heterogeneous group of malignancies derived from the ductal epithelium. The tumor microenvironment (TME) of BC is a complex combination of different cell types and molecules, and it is now considered critical in tumor progression and therapeutic responses (10). Several signal transduction pathways, including Wnt/β/catenin, hedgehog, Notch, BMPs, and PI3K/Akt/NFkB, are deregulated in breast cancer stem cells (CSCs). These signaling pathways stimulate proliferation, migration, invasion, EMT, chemotherapy, and radiotherapy resistance in CSCs. miRNAs also through several signaling pathways can regulate the stemness features and tumorigenesis of CSCs (11). Several studies have demonstrated that CSCs are at the root of tumorigenesis, metastasis, and recurrence, and the behavior of CSCs is highly influenced by their microenvironment (12–16). In recent years, the direct targeting of CSCs has made significant progress (17–21). However, due to the heterogeneity of CSCs and the complex interplay between CSCs and the TME, the translation of this progress into clinical success is still limited. Improvement in the efficacy of BC therapy therefore depends on finding strategies to modulate the immune system in the TME that will reverse or neutralize the suppressive networks within it.

## Upregulation of co-inhibitory molecules

Co-immunosuppressive/co-inhibitory molecules in the TME, including programmed cell death protein 1 (PD-1), cytotoxic T lymphocyte-associated antigen-4 (CTLA-4) (CD152), lymphocyte-activation gene 3 (LAG-3), 2B4, CD160, and TIM-3, can dampen the antitumor mechanism by helping tumor cells escape from host immune surveillance. A significant proportion of BCs express co-inhibitory molecules, and the interaction between the inhibitory receptors and their ligands can be blocked by immune checkpoint inhibitors. Monoclonal antibodies blocking immune checkpoints have already shown potential in clinical trials against multiple solid tumors (22). The United States Food and Drug Administration (FDA) has approved several related drugs that target CTLA-4, PD-1, and its ligand PD-L1, for the treatment of advanced melanoma, non-small cell lung cancer, Hodgkin’s lymphoma, and head and neck cancer (23–27). However, modest results have been observed in BC, where tumors are rarely hypermutated.

Surface-accessible CTLA-4 is largely confined to the TME (28), and its expression is associated with the progression of BC and the effect of therapy. Several studies have indicated that upregulated expression of CTLA-4 enhances BC progression and reduces the therapeutic response (22, 29–31). Ipilimumab was the first immune checkpoint inhibitor approved by the FDA, in 2011, for the treatment of late-stage melanoma. It is a monoclonal antibody that attaches to the CTLA-4 protein receptor to inhibit CTLA-4, and its use has improved 1-year overall survival rates from approximately 35% a decade ago to approximately 95% (32).

It was generally believed that anti-CTLA-4 antibodies caused tumor rejection by promoting the priming of naïve T cells through blocking the inhibitory B7-CTLA-4 signaling in peripheral lymphoid organs. However, this prevailing hypothesis has been questioned in recent years. One study found that CTLA-4 antibodies induced tumor rejection by selective depletion of regulatory T cells (Tregs) in the TME rather than blocking B7-CTLA-4 interaction in the lymphoid organs (12, 33). It has also been observed that some therapies, especially immunotherapy, may lead to immunological changes in the TME (13, 34), and one study found significantly greater protein expression of PD1, PD-L1, and VISTA in prostate tumors after anti-CTLA-4 (ipilimumab) therapy (14, 35). Other studies combining the inhibition of PD-1 and CTLA-4 in solid tumors have highlighted the potential to further enhance the clinical benefits of monotherapies by combining agents with synergistic mechanisms of action (36–38). Preclinical studies also suggest the possibility that radiotherapy can enhance the efficacy of a CTLA-4 blockade in BC (39). One study treated murine melanoma tumor models with a CSC-dendritic cell vaccine, combined with PD-L1 and CTLA-4, and the triple combination treatment significantly enhanced the eradication of CSCs (40). Nolan recently reported that dual checkpoint blockade, anti-PD-1 and anti-CTLA-4, profoundly attenuated the growth of Brca1-deficient BC tumors *in vivo* (41). However, due to the heterogeneity of BC, it has yet to be determined whether different strategies are required to effectively treat different BC subtypes.

In addition to anti-CTLA-4, anti-PD-1/PD-L1 is another immune checkpoint inhibitor that has emerged as an important therapeutic tool in the treatment of cancer in recent years. For the first time pembrolizumab, an anti-PD-1 monoclonal antibody, has been approved in cancer treatments that are based on the tumor biomarkers of high microsatellite instability or mismatch repair deficiency, regardless of the tumor’s original location (42). Tumors with these biomarkers are most commonly found in colorectal, endometrial, and gastrointestinal cancers, but also less commonly appear in cancers arising in places such as the breast, prostate, bladder, and thyroid gland (43–49).

Programmed cell death protein–ligand 1, which is induced in the TME in response to inflammatory signals, is expressed and upregulated in some subtypes of BC, such as triple-negative breast cancer (TNBC), basal BC, and HER2-positive BC (47–51). High PD-L1 expression levels are associated with negative prognostic features such as large tumor size, high grade, lack of estrogen receptor, progesterone receptor, and HER2, and a high proliferative rate (52). However, poor prognosis malignancies with PD-L1 expression may mark cancers as susceptible to PD-1/PD-L1 inhibitor therapies (53–55). Several studies have reported that PD-L1 expression is significantly associated with a better disease-free survival rate in BCs (56, 57). Plasticity of CSCs and heterogeneity in PD-L1 expression profile are factors influencing responses to therapy. Besides, a positive link between dietary lipids with PD-L1 upregulation and increased proportion of CSCs is indicative of the necessity of modifying patient intakes for strengthening the power of immune system against cancer and improving the efficacy of immunotherapy (58). In view of this, immunotherapies combined with PD-1/PD-L1 antibodies have become a potentially curative treatment option for advanced BC. The synergistic effect of anti-PD-1 agent on trastuzumab therapy has been demonstrated in a HER2-positive BC mouse model (59), and another study found that the combination of ado-trastuzumab emtansine (T-DM1) and anti-cytotoxic T-lymphocyte-associated protein 4 (CTLA4) or anti-PD-1 antibodies elicited responses in HER2+ BC xenografts that had previously been resistant to T-DM1 monotherapy (60). While these studies have suggested that a subgroup of patients with BC might benefit from immunotherapy targeting PD-1 and/or PD-L1, the expression of PD-1 on the T cells in the TME and that of PD-L1 on CSCs is not yet well-defined.

## Tumor infiltrating lymphocytes

Tumor infiltrating lymphocytes (TILs) within the tumor environment represent the formation of an immune response to the tumor, and TILs from a patient can be manipulated to be used as treatment for that patient. A previous study demonstrated that the use of a lower dose of interleukin 2 (IL-2) in the context of adoptive cell therapy with TILs was well tolerated and clinically effective in metastatic melanoma patients (23, 61).

There is an accumulation of evidence suggesting that TILs present in BC prior to treatment can predict a positive response to therapy and are associated with an improved prognosis (7, 25–28, 62–64). A meta-analysis study evaluating the predictive role of TILs with respect to neoadjuvant chemotherapy in BC showed that a 10% increment of TILs in stomal or intratumoral sites in a pre-treatment biopsy indicated an increase in the pathologic complete response (pCR) rate (65). Furthermore, several studies reported that lymphocyte predominant BC (involving more than 50% or 60% of lymphocytic infiltration) was associated with an exceptionally high rate of pCR in patients, compared with those without any tumor lymphocytic infiltration (29, 30, 66, 67). Specifically, in a univariate analysis, higher levels of CD8+, CD4+ T, and forkhead box P3+ (FOXP3+) T lymphocytes in the pre-treatment biopsy were correlated with the pCR rate, while CD3+ and lymphocytes in the pre-treatment biopsy were not predictive (65). The tumor cell compartment and the surrounding stromal microenvironment is a subject of continuous modification across the different stages of cancer progression. It is well-known that the microenvironment will change after chemotherapy, as will the TILs subset. It was found that CD3+ and CD8+ infiltrates remained stable during neoadjuvant chemotherapy, while FOXP3+ infiltrate strongly declined (68). The above-mentioned studies indicate that a particular combination of TIL subsets before and after chemotherapy may be a more sensitive predictor for recurrence and survival than a single T lymphocyte type, but more studies are needed to confirm this.

Different subtypes of BC have different levels of TIL infiltrate, and the tumor mutational burden and the presence of TILs are higher among TNBC and HER2+ BCs than other subtypes (69), which clearly correlates with clinical outcomes. In the advanced trastuzumab-resistant HER2-positive BC (known as PANACEA) phase Ib/II study, the efficacy of anti-PD-1 pembrolizumab in combination with trastuzumab was evaluated in metastatic HER2+ BC patients. The results showed that the vast majority of patients had low numbers of TILs in the metastatic niche, even though those with TILs above 5% in the tumor sample were associated with objective response rates (ORR) of 39% vs. 5% in those patients with lower TILs (<5%) (70). In contrast, the pCR of patients with low TILs (0–10%) in TNBC and HER2^+^ BC was not as good as that of patients who manifested high TIL levels at diagnosis (22, 71), suggesting that treatment using these TILs may be an option for these patients. Furthermore, Nadire et al. reported that compared to the HER2-negative BC stem cells, HER2+/CD44+/CD24−/low cells showed a more aggressive phenotype and *in vivo* tumorigenesis with an enhanced resistance to radiation, indicating that HER2-expressing breast cancer stem cells (BCSCs) may be effective targets for the treatment of recurrent tumors (72). The increased recruitment of TILs and their intratumoral expansion, with the combination of IL-2 variant targeting fibroblast activation protein-alpha with anti-HER2 drugs, may help to better position immunotherapy in HER2+ BC in the future. Furthermore, the use of immune-modulating therapeutic approaches to treat other BC subtypes warrants further investigation, and the role that TILs can play is a promising one.

## Target regulatory T cells

Regulatory T cells represent only a minor subset of CD4+ T cells and appear to play an important role in cancer immunology (73). Previous work has clearly established that Tregs are increased in most human solid tumors, and the accumulation of Tregs in the TME may prevent the protective antitumor immunity of immune cells and the optimal functioning of the TME. More importantly, Tregs at a tumor site overexpress inhibitory receptors CTLA-4, PD-1, TIM-3, and LAG-3 and up-regulate expression of TGF-β-associated LAP and GARP molecules and NRP-1 (32, 74), further enhancing their capability to suppress antitumor functions and, thus, contributing to tumor escape from the host immune system.

As an important component of the TME, Tregs are involved in regulating the stemness of tumor cells, and they are associated with CSCs in BC. One study found that CD4+ CD25+ Tregs increased the aldehyde dehydrogenase (ALDH)+ population of mouse BC cells, promoted their sphere formation, and enhanced the expression of stemness gene sex determining region Y-box 2 (Sox2). On the other hand, Sox2-overexpression tumor cells activated NF-κB-CCL1 signaling to recruit Tregs (75). Another study reported that Oct4^high^ breast CSCs could interact with MSCs to polarize the T-cell response, in which CD4+, CD25+, and FOXP3+ Tregs increased while Th17 decreased. This interaction required C-X-C chemokine receptor type 4 and connexin 43-dependant gap junctional intercellular communication (GJIC). Since GJIC between CSCs and MSCs allows the CSCs to maintain dormancy (76), the interaction will allow the cancer cells to survive and establish dormancy, which may be the main reason for recurrence (77). These findings reveal the functional interaction between Tregs and CSCs and indicate that targeting the communication between them is a promising strategy in BC therapy.

Another recent study analyzed the features of 100 patients with untreated BC and reported that FOXP3+ Treg cells accumulating in tumor sites were associated with aggressive BC phenotypes, such as TNBC, and also correlated with higher grade lesions across all subsets (78). All the relevant studies have indicated that the elimination or silencing of Tregs could be a desirable therapeutic objective for BC and that surface molecules expressed by Tregs can be specifically targeted by (daclizumab: anti-CD25 Ab) Abs or pharmacological inhibitors. To date, a variety of agents, including Abs and IL-2 fusion toxins such as denileukin diftitox (Ontak), or drugs, such as cyclophosphamide or tyrosine kinase inhibitors (sunitinib), have been tested in preclinical *in vitro* studies with human cells (79–84). Some studies reported the efficiency in depleting Tregs and the tolerance of the above drugs in patients with cancer, and others reported a boosted immune response (4, 5). A clinical trial of daclizumab was performed in patients with metastatic BC, in combination with an experimental cancer vaccine, and robust CD8+ and CD4+ T cell priming and the boosting of vaccine antigens were observed (6). However, to date, obvious clinical benefits have not been observed in patients who received Tregs depletion treatment. This may be due to the inadequate depletion efficacy of the drugs, the innate resistance of Treg to certain drugs, the selective sensitivity of some but not all Treg subsets to the drugs being used, or the ability of the host to rapidly re-populate the depleted Treg. More selective strategies that eliminate only those that mediate the suppression of antitumor immunity are needed. In 2016, Plitas et al. found that CCR8 was differentially expressed by the entire tumor–resident Treg cell population, which indicated that targeting CCR8 may be a promising means by which to selectively deplete Tregs in the TME, although the role of CCR8 in Treg function remains unclear (78). More markers specific to human immunosuppressive Tregs are yet to be defined.

## Targeting breast cancer stem cells directly

Preclinical and clinical research has indicated that conventional chemotherapy and endocrine treatment lead to the significant enrichment of BCSCs and eventually contribute to drug resistance (10, 12, 13). Though the exact origin of BCSCs on five molecular subtypes of BC determined by gene expression profiling has stirred much controversy (14), the immunoresistance imposed by BCSCs has undoubtedly been one of the major mechanisms responsible for treatment failure. It would be a highly promising strategy to specifically target BCSCs with immunotherapy to lessen immunoresistance and eradicate the roots of BC. The next generation agents for this cellular compartment of BC are being developed to directly target specific antigen, protein, and immune inhibitory molecules that govern the stemness as well as the fate of BCSCs (summarized in **Figure 1**).

## Tumor associated antigen-targeted vaccines

As the most potent antigen-presenting cells, dendritic cells (DCs) are widely used as tools for anticancer vaccination. For example, DCs pulsed with CD44 peptide effectively killed human BCSCs *in vitro* by enhancing T cell stimulation and generating potent cytotoxic T lymphocytes (CTL) (15). Since CSC lysates contain all the antigens responsible for stemness, theoretically, it is an ideal antigen source for vaccine generation. In 2012, a CSC lysate-DC vaccine was first reported to induce significant protective immunity in melanoma and head and neck cancer *in vivo* (16). Afterwards, Pham et al. demonstrated that BCSC lysate-pulsed DC vaccine lengthened the survival of BC humanized mice (17). This is supported by the unpublished data of the authors, which showed that 4T1 ALDH^high^ BCSC lysate-DC vaccine enhanced the killing capacity of CTLs with regard to ALDH^high^ 4T1 BCSCs *in vitro* and reduced lung metastasis *in vivo*. These results suggested its efficacy should be investigated in a clinical trial. A phase I/II clinical trial in China evaluated the safety of ALDH^high^ CSCs lysates-DC vaccine in metastatic BC (NCT02063893), but the result has still not been published.

In addition to ALDH being a CSC marker, inhibiting ALDH activity was found to block irradiation-induced stemness and decrease breast tumor growth and metastasis (18), which provided a rationale for utilizing it as a target for immunotherapy. The stimulation of CD8^+^ T cells with ALDH1A1 peptide-pulsed APC recognized and eliminated the ALDH^high^ CSCs and inhibited tumor growth and metastases in various solid tumors, including BC (19). The authors’ ongoing study shows that an ALDH 1A1+1A3 peptide-DC vaccine elicits significantly stronger T cell and B cell immune responses against the tumor, compared with an ALDH1A1 or ALDH1A3 peptide-DC vaccine alone.

Novel vaccine carriers are now emerging, and, in one study, stage-specific embryonic antigen 4 (SSEA4) was specifically overexpressed on BC cells and BCSCs, and carbohydrate-based and SSEA4-targeted vaccines combining a glycolipid adjuvant showed induced immunoglobulin G (IgG) antibodies specifically bound to SSEA4 and its tetrasaccharide epitope in BC (20). It is widely acknowledged that induced pluripotent stem cells (iPSCs) can be used as immunization agents to target CSCs by promoting an anti-tumor response due to overlapping antigens between iPSCs and cancer cells. A recent report showed that an autologous iPSC/oligodeoxynucleotide vaccine mounted strong B and T Cell responses against epitopes and prevented tumor growth in syngeneic murine DB7 BC, mesothelioma, and melanoma models (21), indicating that a CSC vaccine could be used for cancer treatment.

## Human epidermal growth factor receptor 2-targeted immunotherapy

A significant body of evidence has accumulated to support the notion that HER2 drives tumorigenesis, invasion, and treatment resistance by regulating BCSCs, even in BCs that do not display HER2 gene amplification (22–27) Thus, HER2-targeted immunotherapy can contribute to therapeutic efficacy by directly eliminating BCSCs, especially in TNBC.

More than 60 clinical trials have focused on a HER2-targeted vaccine. The most studied immunogenic peptide is E75 (nelipepimut-S), an HLA-A2- and HLA-A3-binding 9 amino acid peptide derived from HER2. It has been used in multiple vaccine formulations by means of loading it on to autologous DCs, or embedding it in longer peptides capable of enhancing T-cell responses, and combining it with various immunoadjuvants (28). Early in 2000, in a pilot study, Brossart et al. suggested that E75-pulsed DCs can induce antigen-specific CTL responses in heavily pretreated patients with BC (29). The final report from a phase I/II clinical trial of the E75 vaccine showed that it statistically increased the 5-year disease-free survival in patients with BC who had completed a standard-of-care therapy (n = 108) compared with those who did not receive a vaccine (n = 79) (89.7% vs. 80.2%, P = 0.08), while local and systemic toxicities were mild (28). Unlike the E75-pulsed DC vaccine, another HER2-derived peptide (AE37) pulsed-DC vaccine aimed to primarily elicit the CD4^+^ T cell response, not the CD8^+^ T cell response, and was shown to be safe and capable of generating durable immune responses and ultimately preventing BC recurrence in a clinical trial (30). Apart from the use of vaccines in the adjuvant and metastatic setting, it would be feasible to assess the efficacy of a vaccine as a neoadjuvant treatment when it is administered prior to surgery. Sharma et al. demonstrated that 90% (9/10) of patients with breast ductal carcinoma *in situ* with HER2-positive subtypes demonstrated a clinical response to the 6 HER2/neu MHC class II promiscuous-binding peptide-pulsed DC vaccine, accompanied by a decline and/or eradication of HER2/neu expression in some patients (31). Of these clinical trials involving HER2-targeted vaccines, only one three-phase trial related to E75 has been completed, but the results have not been published yet. Furthermore, as a new vaccine carrier, the adeno-associated virus expressing specific HER2-peptide delayed the growth of the tumor in D2F2/E2 bearing BALB/C mice by establishing an active immune response (22). And HER2- virus-like particle vaccine showed promise as a new cost-effective modality for prevention and treatment of HER2-positive cancer by reducing spontaneous development of mammary carcinomas by 50%-100% in human HER2 transgenic mice and inhibited the growth of HER2-positive tumors implanted in wild-type mice (85).

Despite the fact that trastuzumab-based treatment has attractive clinical benefits, in one study, 70% of HER2-positive BCs showed primary resistance to trastuzumab, with the majority of patients developing secondary resistance during 1–2 years of treatment (32), which promoted the development and approval of an antibody-drug conjugate (ADC) and/or anti-HER2 bispecific antibody (HER2Bi), such as trastuzumab-emtansine. MEDI4276 is an investigational ADC being developed by MedImmune, and it is characterized by the anti-HER2 antibody backbone conjugated with the cytotoxic anti-microtubule agent tubulysin. The unpublished data shows that MEDI4276 can inhibit D2F2/E2 tumor growth and reduce the number of HER2-positive cells and ALDH^high^ CSCs by inducing significant host immune responses of CD3^+^ and CD19^+^ TILs in mouse breast tumor D2/F2 and TNBC 4T1, with enforced expression of HER2, D2F2/E2, and HER2-4T1. Novel HER2Bis, targeting the HER2/Her-3 or CD3/HER2, are being developed with encouraging preclinical or early phase clinical results (32). Fox example, MM-111, a bispecific HER2/Her-3 antibody fusion protein, has been developed to overcome the Her-3-mediated resistance to currently existing anti-HER2 therapies (NCT01304784). Moreover, a dynamic model of HER2 status was developed for the detection of druggable targets that may counteract resistance to HER2-targeted therapy due to HER2 loss and have identified PDGFR-B as a possible target and proved the ability of sunitinib in delaying growth of tumors that evolved from HER2-positive to HER2-negative status (86).

Adoptive T-cell therapy has been one of the most exciting fields of immunotherapy in recent years. Lum et al. have done a great deal of work on activated T cells (ATC) armed with CD3 and HER2-targeted bispecific antibody and found they exhibited high levels of specific cytotoxicity in breast and prostate cancer. A phase I trial by the same team demonstrated that eight infusions of HER2Bi-armed ATC, in combination with low dose IL-2 and granulocyte-macrophage colony-stimulating factor, induced anti-tumor responses and increases in Th1 cytokines and IL-12 serum levels, without dose-limiting toxicities, in patients with HER2-positive and negative BC (33). The repeated infusions of armed ATC may help to overcome the tumor immunosuppressive factors and recruit endogenous immune cells leading to *in situ* vaccination (33). Moreover, one ongoing study by the authors has indicated that mouse tumor-draining lymph node T cells armed with anti-mCD3/anti-m HER2 bi-specific antibody may selectively target HER2-positive BCSCs and, thus, prevent metastasis in a mouse 4T1 xenograft that does not have HER2 gene amplification, but they have failed to inhibit the HER2-negative primary tumor. Interestingly, such T cells have prevented metastasis and significantly inhibited local tumor growth by targeting both CSCs and non-CSCs in a mouse HER2-4T1 model. A phase II study is estimating the effect of HER2Bi-armed ATC in women with stage II–III TNBC without a complete pathologic response who are receiving a regimen of neoadjuvant chemotherapy, surgery, and/or irradiation (NCT01147016). Compared with all above HER-targeted therapy, M.Moasser believed that to inactivate the driving HER2 oncogene, remains a holy grail with a potential that greatly exceeds all current HER2 targeting therapeutics (87).

## Immune checkpoint inhibitors and other new emerging antigens

CSCs showed a high expression of PD-L1 in BC, colon cancer, and glioma (34, 35), and there was a bidirectional effect between the epithelial-mesenchymal transition status and PD-L1 expression, especially in cells in the claudin-low subtype of BC (36). Almozyan et al. found a novel role for PD-L1 in sustaining the stemness of BC cells in the most immunocompromised mouse model (NOD/SCID/IL-2R^-/-^) (37). Another unpublished study by the authors shows that CSCs can both directly and indirectly inhibit B cell function through the PD-L1/PD-1 axis on both B cells and Th cells, and anti-PD-L1 could paralyze the suppression of BCSCs on the IgG production secreted by 4T1 tumor reactive B cells. These results suggest that the PD-1-PD-L1/PD-L2 signaling axis plays an important role in CSCs-driven tumor immune resistance. Thus, immunologically targeting CSCs while simultaneously blocking PD-1/PD-L1 and/or CTLA-4-mediated immune suppression may significantly enhance the outcome of current cancer immunotherapies (38).

Integrin β4 is involved in tumor formation, invasion, and metastasis in BC, and it could be a potential antigen for designing novel immunotherapies. One more ongoing study by the authors has shown that an integrin β4 protein-pulsed DC vaccine can significantly kill ALDH^high^β4^high^ 4T1 CSCs and inhibit tumor growth in a Balb/c mouse 4T1 xenograft model. In addition, CD3 and integrin β4-targeted bi-specific antibody have demonstrated a significant anti-tumor effect on this 4T1 model. Transcription factors Oct-4, Sox2, and nanog are all key regulators of embryonic stem cell maintenance. Sox2 was identified as a novel antigen in glioma, and targeting a Sox2 vaccine successfully improved glioma T-cell-based immunotherapy (36). Furthermore, Sox2, Oct-4, and nanog, mediating tumorigenesis and metastasis, were upregulated in tamoxifen‐resistant cells and in patients who did not respond to tamoxifen treatment (40–42). Thus, the next generation immunotherapies targeting BCSCs transcription factors, such as vaccines and specific antibodies, represent a promising hope with regard to immunosuppression and overcoming treatment resistance in BC.

## Opportunities to further boost the anti-breast cancer immune response

Although stunning successes with cancer immunotherapy have been achieved with respect to melanoma, lung cancer, and other malignancies, only modest results have been observed for the relatively immunological cold breast tumors. The ORRs of PD-L1 or PD-1 monoclonal antibody treatment are in the 12%–19% range (10, 12, 13), while CTLA-4 blockade appears minimally active (14) Therefore, advances in immune treatment strategies that can further boost the anti-BC immune response by altering the immune-suppressive TME are needed.

## Combined immunotherapy

Although clinical trials have validatedmmune-oncology as a new pillar of anticancer therapy, there is still tremendous potential for synergistic combinations of immunotherapy agents and for combining immunotherapy agents with conventional cancer treatments.

The combining blockade of CTLA-4 and PD-1/PD-L1 is synergistic and is of clinical benefit, and it may serve as a paradigm to guide future approaches to immune-oncology combination therapy. Meanwhile, conventional cancer therapies, such as chemotherapy and radiotherapy, and targeted therapy can not only kill tumor cells, but they also have an effect on the different components of the immune system, suggesting a potentially synergistic benefit of combining these therapies with immunotherapy (15, 16). In a BRCA1-mutant BC mice model, Nolan et al. found that cisplatin was required for a treatment response to checkpoint blockade, since no attenuation in tumor growth was observed with combined anti–PD-1 and anti-CTLA4 therapy alone (41). Very recently, a phase III study of anti-PD-L1 (atezolizumab) and nab-paclitaxel in advanced triple-negative BC showed that, among patients with PD-L1 positive tumors, progression-free survival was statistically significantly longer in patients treated with the combination of atezolizumab + nab-paclitaxel compared with those treated with a placebo + nab-paclitaxel (17). An FDA decision regarding the approval of combination therapy for certain types of BC is anticipated by 12 March 2019. If approved, the chemo combination would become the first cancer immunotherapy regimen indicated for the treatment of PD-L1-positive metastatic triple-negative BC.

The use of vaccines is an attractive strategy for the prevention of BC relapse in patients without measurable cancer but who have a high chance of recurrence. Two types of vaccine strategies are being tested in patients with BC to prevent recurrence: cell-based vaccines and BC antigen-specific vaccines. However, two vaccination strategies (HER2 peptides and sialyl-Tn- keyhole limpet haemocyanin) that showed promise in the early phase of testing disappointingly failed to meet the primary clinical endpoints in randomized studies (17, 18). Strategies combining checkpoint blocking antibodies and vaccination have a high potential, but although impressive clinical results have been obtained with adoptive cell therapy in hematological malignancies, progress in using chimeric antigen receptor-T cells to treat BC has been limited.

## Conclusion

This review focuses on the development of immunoresistance in BCs and discusses immunotherapies to treat refractory BC by using strategies that directly modulate the immune system and BCSCs. Such strategies include the combination of two checkpoint blocking antibodies, the addition of checkpoint blocking antibodies to traditional chemotherapy or radiotherapy, or vaccines.

## Author contributions

Conception and design of the research: ML. Obtaining financing: LP. Writing of the manuscript: LP and JH. Critical revision of the manuscript for intellectual content: ML. All authors contributed to the article and approved the submitted version.
